# Cooperative and independent functions of FGF and Wnt signaling during early inner ear development

**DOI:** 10.1186/s12861-015-0083-8

**Published:** 2015-10-06

**Authors:** Kevin D. Wright, Amanda A. Mahoney Rogers, Jian Zhang, Katherine Shim

**Affiliations:** Department of Pediatrics, Children’s Research Institute, Medical College of Wisconsin, Milwaukee, WI 53226 USA

**Keywords:** Inner ear, Otic placode, Sprouty1, Sprouty2, Wnt8a, FGF, β-catenin, Cross talk

## Abstract

**Background:**

In multiple vertebrate organisms, including chick, *Xenopus*, and zebrafish, Fibroblast Growth Factor (FGF) and Wnt signaling cooperate during formation of the otic placode. However, in the mouse, although FGF signaling induces *Wnt8a* expression during induction of the otic placode, it is unclear whether these two signaling pathways functionally cooperate. Sprouty (*Spry*) genes encode intracellular antagonists of receptor tyrosine kinase signaling, including FGF signaling. We previously demonstrated that the *Sprouty1* (*Spry1*) and *Sprouty2* (*Spry2*) genes antagonize FGF signaling during induction of the otic placode. Here, we investigate cross talk between FGF/SPRY and Wnt signaling during otic placode induction and assess whether these two signaling pathways functionally cooperate during early inner ear development in the mouse.

**Methods:**

Embryos were generated carrying combinations of a *Spry1* null allele, *Spry2* null allele, *β-catenin* null allele, or a Wnt reporter transgene. Otic phenotypes were assessed by in situ hybridization, semi-quantitative reverse transcriptase PCR, immunohistochemistry, and morphometric analysis of sectioned tissue.

**Results:**

Comparison of *Spry1*, *Spry2*, and Wnt reporter expression in pre-otic and otic placode cells indicates that FGF signaling precedes and is active in more cells than Wnt signaling. We provide in vivo evidence that FGF signaling activates the Wnt signaling pathway upstream of TCF/Lef transcriptional activation. FGF regulation of Wnt signaling is functional, since early inner ear defects in *Spry1* and *Spry2* compound mutant embryos can be genetically rescued by reducing the activity of the Wnt signaling pathway. Interestingly, we find that although the entire otic placode increases in size in *Spry1* and *Spry2* compound mutant embryos, the size of the Wnt-reporter-positive domain does not increase to the same extent as the Wnt-reporter-negative domain.

**Conclusions:**

This study provides genetic evidence that FGF and Wnt signaling cooperate during early inner ear development in the mouse. Furthermore, our data suggest that although specification of the otic placode may be globally regulated by FGF signaling, otic specification of cells in which both FGF and Wnt signaling are active may be more tightly regulated.

**Electronic supplementary material:**

The online version of this article (doi:10.1186/s12861-015-0083-8) contains supplementary material, which is available to authorized users.

## Background

The inner ears, embedded on each side of the skull, are the organs responsible for the detection of sound, linear acceleration, and rotational movement. Most of the cells that compose each inner ear and associated cochleovestibular ganglion are derived from the otic placode, a thickened region of embryonic ectoderm located lateral to each side of the developing hindbrain [1–8]. Each otic placode invaginates to form an otic cup, pinches off from the surface ectoderm to form an otic vesicle, and undergoes complex morphogenesis to form the mature inner ear. Multiple extracellular signals, including Fibroblast Growth Factor (FGF) and Wnt, are required for formation of the otic placode [2, 4–6]. However, the cooperative vs. distinct roles of these pathways during otic placode induction and patterning in mammals are not clear.

All cranial placodes, including the otic placode, originate from the pan-placodal region (PPR), a U-shaped, ectodermal domain located adjacent to the anterior neural plate and neural crest during gastrulation (reviewed in [1, 2, 9, 10]). In multiple organisms, FGF signaling is required to specify the posterior PPR as the domain from which the otic and epibranchial placode cells will segregate, called the otic-epibranchial progenitor domain (OEPD, reviewed in [2, 6, 8, 11–13]). In contrast, Wnt signaling is required to stabilize otic vs. epidermal/epibranchial cell fate decisions within the OEPD [11, 14–16]. In mouse, the Wnt signaling reporter, TCF/Lef-lacZ [14, 17], is active after specification of the OEPD [14, 18, 19]. Furthermore, in chick, either activation or inhibition of Wnt signaling has no affect on formation of the OEPD [15, 18]. In zebrafish, heterogeneous levels of *pax2a* or *pax8* transcript and/or protein are observed in the ectoderm prior to otic placode formation, and high levels of *pax2a* or *pax8* expression favor otic differentiation over epibranchial fates [16, 20]. Whereas FGF signaling is required for specification of the number of Pax2a + progenitor cells, the distribution of high vs. low-expressing Pax2a + cells is dependent on Wnt signaling activity [16, 18]. These data are consistent with the initiation of FGF signaling during induction of the OEPD, and the initiation of Wnt signaling later during the specification and patterning of otic placode cells within the OEPD.

Consistent with a role of Wnt signaling in otic fate decisions, otic vesicles are reduced in size when Wnt signaling is inhibited [14, 15, 21, 22]. Early ectopic activation of Wnt signaling results in the formation of enlarged or supernumerary otic vesicles due to posteriorization of the embryo leading to ectopic expression of both FGF and ectodermally-expressed otic competence factors, such as Foxi1 [21, 23, 24]. Later activation of Wnt signaling in the OEPD by expression of an activated form of the Wnt signaling effector, β-catenin, in Pax2-expressing cells in the mouse results in the expansion of otic cells at the expense of neighboring epidermal/epibranchial cells [14]. Enlarged otic vesicles are also observed in zebrafish embryos treated during somitogenesis with a chemical activator of Wnt signaling, BIO, which inhibits glycogen synthase kinase 3β (GSK3β)-mediated degradation of β-catenin [16]. Similarly, enlarged or ectopic otic vesicles are observed in gain-of-function experiments in which Fgf is overexpressed in multiple organisms (reviewed in [13], see also [25]). However, misexpression of Fgf can also lead to an opposite phenotype – the inhibition of otic differentiation [15, 25, 26]. Combined, these data indicate that the timing, dosage and spatial distribution of both FGF and Wnt signaling during the induction of the otic placode must be tightly regulated [15, 16, 21, 25, 26].

In multiple organisms, FGF and Wnt signaling cooperate during induction of the otic placode. In chick, Wnt8c functions synergistically with FGF signaling to induce otic fate [27]. In *Xenopus*, whereas inhibition of either FGF or Wnt signaling alone reduces the ability of neural plate tissue to induce the expression of the otic marker, *pax8*, in explant cultures, simultaneous inhibition of both FGF and Wnt signaling almost completely abrogates *pax8* induction [28]. Furthermore, morpholino (MO) knockdown of combinations of Wnt and Fgf genes (*eg.* Fgf8-MO + Wnt8-MO) in *Xenopus* results in greater reduction of otic expression of *pax8* and *sox9* than single morpholino knock-down of Fgf or Wnt alone [28]. Similarly, in zebrafish, morpholino knock-down of *wnt8b* in *fgf3* mutant embryos produces otic vesicles that are smaller than otic vesicles produced after inactivation of either *wnt8b* or *fgf3* alone [22].

In mice, expression of *Wnt8a* in the hindbrain is reduced in *Fgf3*^*−/−*^*; Fgf10*^*−/−*^ double mutant embryos and otic vesicles are reduced or absent [29]. Sprouty genes encode antagonists of receptor tyrosine kinase (RTK) signaling, including FGF signaling. We have previously demonstrated that in *Spry1*^*−/−*^*; Spry2*^*−/−*^ double mutant embryos, the otic placode is larger and *Wnt8a* expression is expanded [18]. Combined, these data demonstrate that FGF signaling regulates the Wnt pathway at the level of *Wnt8a* expression. However, recent data demonstrate that otic placodes form normally in *Wnt8a*^*−/−*^ knockout embryos [30]. Furthermore, Vendrell et al. demonstrate that the otic placode and vesicle form normally upon combinatorial inactivation of *Wnt8a* and either *Fgf3* or *Fgf8* (*Wnt8a*^*−/−*^*; Fgf3*^*−/−*^ or *Wnt8a*^*−/−*^*; Fgf8*^*flox/d2,3*^*; Mesp1Cre/+* embryos). Thus, there is currently little evidence in mammals that cross talk between FGF and Wnt signaling is functionally required during otic placode induction.

Inactivation of *Spry* genes results in over-activity of receptor tyrosine kinase signaling in its normal tissue context. Here, we took advantage of the enlarged placode in *Spry1*^*−/−*^*; Spry2*^*−/−*^ mutants to assess expression of Wnt pathway components and Wnt signaling activity in tissues in which FGF-response is elevated. This allowed us to further characterize the relationship between FGF and Wnt signaling during otic placode induction and to assess whether these two signaling pathways functionally cooperate during early mammalian inner ear development.

## Methods

### Mouse lines

Mouse lines carrying null or floxed alleles of *Spry1* [31], *Spry2* [32], the conditional *β-catenin*^*flox/flox*^ allele ([33], purchased from the Jackson Laboratory, B6.129-*Ctnnb1*^*tm2Kem*^/KnwJ, stock number 004152), *β-actin-Cre* [34] and TCF/Lef-lacZ reporter mice [17] were maintained and genotyped as described. *Spry* mutant embryos were generated by crossing *β-actin cre/β-actin cre*; *Spry1*^*−/+*^; *Spry2*^*−/+*^ males to *Spry1*^*flox/flox*^; *Spry2*^*flox/flox*^ females. For all experiments in which *Spry1*^*−/+*^; *Spry2*^*−/+*^ mutant embryos were used as littermate control embryos, comparison was made to CD-1 embryos to verify the absence of defects. Wnt reporter activity was assessed in *Spry*-deficient embryos by crossing *β-actin cre/β-actin cre*; *Spry1*^*−/+*^; *Spry2*^*−/+*^ males to *Spry1*^*flox/flox*^; *Spry2*^*flox/flox*^; *TCF/Lef-lacZ/+* or *Spry1*^*flox/flox*^; *Spry2*^*flox/flox*^; *TCF/Lef-lacZ/TCF/Lef-lacZ* females. *β-catenin*^*−/+*^ animals were generated by crossing *β-catenin*^*flox/flox*^ mice to *β-actin-Cre*/*β-actin-Cre* animals. Resulting progeny were bred to both remove the *β-actin-Cre* allele and to generate *Spry1*^*flox/flox*^; *Spry2*^*flox/flox*^; *β-catenin*^*−/+*^ animals. Genetic interaction experiments were performed by generating embryos from a *β-actin cre/β-actin cre*; *Spry1*^*−/+*^; *Spry2*^*−/+*^ male to *Spry1*^*flox/flox*^; *Spry2*^*flox/flox*^; *β-catenin*^*−/+*^ female cross. CD-1 embryos were used as *Spry*^*+/+*^; *Spry2*^*+/+*^ controls to assess the size of the *Wnt8a* expression domain. All procedures were approved by the Institutional Animal Care and Use Committee at the Medical College of Wisconsin.

### In situ hybridization

Embryos were staged so that noon on the day of vaginal plug detection was designated as embryonic day (E) 0.5. Embryos were dissected in phosphate buffered saline, 0.1 % Tween-20 and fixed by immersion in 4 % paraformaldehyde at 4 °C for one hour. Whole-mount in situ hybridization was performed according to standard protocols, using the following digoxigenin-labeled probes: *Spry1*, *Spry2*, *Pax8, Foxi2, Wnt8a, Wnt1, Wnt3a, Wnt6, Fzd1,* and *Fzd8*. All probes were tested on CD-1 wild-type embryos, and expression patterns were compared to published images. Scoring of changes in gene expression patterns or levels was based upon comparison of all embryos of a particular genotype with all embryos of another genotype. Photography of whole-mount embryos was done at the same exposure, using a Zeiss Discovery V.12 microscope.

### Microdissection and semi-quantitative reverse transcriptase PCR

OEPD, underlying mesenchyme, and adjacent neural ectoderm were microdissected from 5–7 s embryos using tungsten needles and fine forceps [29]. Tissues from each embryo were stored separately in TRIzol reagent (Life Technologies) at −80 °C before genotyping. Tissues from 2 embryos of the same genotype were pooled. RNA was isolated and reverse-transcribed using the SuperscriptIII first-strand synthesis kit and oligo-dT primers (Life Technologies). For each gene, primers were validated for specificity by inclusion of “no reverse transcriptase” samples and ability to amplify a single band of the correct size. For each primer pair, test PCRs were performed across a series of cycle numbers to identify the linear range prior to plateau [35]. Experimental PCRs were performed at a cycle number in the linear range using GoTaq 2X polymerase (Promega). All PCR reactions were performed at least twice on 2–3 biological replicates (4–6 embryos). *Gapdh* was used as a pipetting control.

Primer sequences were: *Gapdh* forward 5′- GGGCTGGCATTGCTCTCAATGACAACTTT-3′ and reverse 5′- CACCCTGTTGCTGTAGCCGTATTCAT-3′; *Wnt1* forward 5′-CAGCTGGGTTTCTACTACGTT′3′ and reverse 5′-CAGACTCTTGGAATCCGTCAA-3′; *Wnt3a* forward 5′-TGCCATGAACCGTCACAA-3′ and reverse 5′-CAGCAGGTCTTCACTTCACA-3′; *Wnt6* forward 5′- GCCAGACTGCGGTAGAG-3′ and reverse 5′- GTAGGATCCATGACCAAGGG-3′; *Wnt8a* forward 5′-GGTGGAATTGTCCTGAGCAT-3′ and reverse 5′-GTTCTTGGTGACTGCGTACA; *Wnt3* forward 5′-GCCAAGAGTGTATTCGCATCTA-3′ and reverse 5′-TCATGGGACTTCGATGAATGG-3′; *Wnt5a* forward 5′-TGGCAGGGTGATGCAAATA-3′ and reverse 5′-CTGCAGCCACAGGTAGAC-3′; *Wnt5b* forward 5′- CGAGAGCGTGAGAAGAACTTT-3′ and reverse 5′- GGCGACATCAGCCATCTTAT-3′; *Wnt7b* forward 5′- GGATGCCCGTGAGATCAAA-3′ and reverse 5′- GACACACCGTGACACTTACA-3′; *Fzd3* forward 5′- GCTTTGAATGGGCCAGTTT-3′ and reverse 5′- TCAGGAGTGACTGAGCAAAG-3′; *Fzd7* forward 5′- AAAGGCAGTGGCCGAAA-3′ and reverse 5′- TCTCTCTCTGCTGGTCTCAA-3′.

### LacZ stain

To detect β-galactosidase activity in embryos carrying the TCF/Lef-lacZ transgene, embryos were dissected in phosphate buffered saline, 0.1 % Tween-20, then fixed and washed. Embryos were incubated with X-gal as described in [17] except that incubations were performed at room temperature for varying lengths of time. All OEPD-stage embryos and otic placode-stage embryos that were sectioned for measurement analysis (Fig. 4e, f) were incubated in X-gal overnight. To assess differences in intensity of staining at otic placode stages, embryos were incubated in X-gal for 60 mins. (Additional file 2: Figure S2) or 90 mins. (Additional file 1: Figure S1). Whole-mount embryos were photographed using a Zeiss Discovery V.12 microscope.

To compare *Spry1*, *Spry2*, and TCF/Lef-lacZ reporter expression domains, embryos were stained in whole-mount, post-fixed overnight in 4 % paraformaldehyde, then embedded in JB-4 plastic (Polysciences). Serial transverse sections were cut at 6 μm thickness. To compare staining patterns between embryos stained for different markers, sections were aligned by morphological criteria. The posterior PPR was identified as the ectoderm present in sections in which the notochord was visible and the intra-embryonic coelomic cavity had split into two left and right horns. At the OEPD stage, the anterior end was designated as the start of the appearance of cubiodal vs. squamous epithelium and the posterior end of the OEPD was designated at the transition from cubiodal to squamous epithelium. Sections containing the OEPD coincided with the presence of the first branchial pouch and OEPD was absent in sections where the entrance to the foregut diverticulum was visible. The otic placode was defined based upon the presence of a thickened pseudostratified epithelium, at least two nuclei thick.

### E-cadherin stain

For E-cadherin immunostaining, embryos were dissected at E9.0 to E9.5 and were stained in whole mount with anti-E-Cadherin antibody (Life Technologies, 1:1000 dilution). A biotinylated anti-rat IgG secondary antibody (Vector Laboratories), followed by Vectastain Elite ABC (Vector Laboratories) and TSA fluorescein tyramide reagent (PerkinElmer) amplification, were used to detect anti-E-cadherin as described [36]. Embryos were photographed using a Zeiss Observer Z1 inverted microscope.

To compare E-cadherin staining intensity, mutant and control embryos were fixed in 4 % paraformaldehyde at 4 °C overnight, cut by vibratome, then stained with anti-E-cadherin antibody (Life Technologies, 1:1000 dilution), using an Alexa-488-conjugated anti-rat IgG secondary antibody (Life Technologies, 1:1000 dilution) as described [37]. Images were collected using a Zeiss LSM510 laser scanning confocal microscope (data not shown).

### Morphometric analysis

To measure the size of the otic placode, reporter-positive, and reporter-negative domains, embryos were stained for reporter activity in whole-mount, then embedded in JB-4 plastic (Polysciences). Serial transverse, 6 μm-thick sections were cut. Embryos in which more than 4 sections were lost during microtomy were excluded. All sections containing the otic placode were photographed, and images were used to measure the medial-to-lateral length of the otic placode using Image J software. The otic placode was identified based on its pseudostratified epithelial morphology, and was measured if it was at least two nuclei thick. Using Image J, a freehand line was drawn along the basal surface of the otic epithelium, following the curvature of the otic placode. For the majority of embryos, both the left and right side of the embryos were measured.

For the total placode, reporter-positive, and reporter-negative domains, average cross-sectional basal surface areas (mm^2^) were calculated as the sum of the medial-to-lateral lengths from all sections multiplied by 0.006 mm (the thickness of each section). Fold area changes were calculated by normalizing the area for each placode by the mean area in *Spry1*^*−/+*^*; Spry2*^*−/+*^ controls. Significance of difference between genotypes was measured by one-way analysis of variance (ANOVA).

Medial-to-lateral, total placode measurements were graphed by aligning from the widest length, which was centered on “0” at the y-intercept. Measurements from each individual placode were graphed, along with average measurements. Start and end of the averaged plot were determined by calculating the average anterior and posterior distances from the y-intercept. Medial-to-lateral reporter-positive and reporter-negative measurements were graphed to align with the total placode measurements. Therefore, all graphs were aligned by the maximal width of the total placode.

Anterior-posterior lengths of *Spry1*^*−/+*^*; Spry2*^*−/+*^*; β-catenin*^*+/+*^ embryos, *Spry1*^*−/−*^*; Spry2*^*−/−*^*; β-catenin*^*+/+*^ embryos, and *Spry1*^*−/−*^*; Spry2*^*−/−*^*; β-catenin*^*−/+*^ embryos were measured by identifying the number of transverse sections containing an otic placode and multiplying by the thickness of each section (0.01 mm). Otic placodes were identified as thickened epithelium, at least 2 cells thick, that did not express *Foxi2*.

## Results

### *Spry1* and *Spry2* were expressed earlier and in a broader domain than a Wnt reporter

We previously demonstrated that *Spry1* and *Spry2* were expressed in the OEPD ectoderm and underlying mesenchyme, and that expression of both genes restricted to the ectoderm when the otic placode was morphologically distinct [18]. Groves and colleagues have demonstrated that the Wnt signaling reporter, TCF/Lef-lacZ [17], is active after induction of the OEPD, marked by *Pax2* expression [14, 19]. Furthermore, the TCF/Lef-lacZ reporter is expressed in anterior and medial regions of the otic placode, but is absent from a posterior-ventrolateral domain [14]. To define the spatial and temporal relationship between *Spry1* and *Spry2* expression and Wnt signaling activity, we re-examined *Spry1* and *Spry2* expression by in situ hybridization to 1) determine the timing of onset of expression of these genes in the PPR or OEPD and 2) determine whether these genes were expressed in posterior regions of the OEPD and otic placode, where TCF/Lef-lacZ reporter activity is absent.

**Fig. 1:**
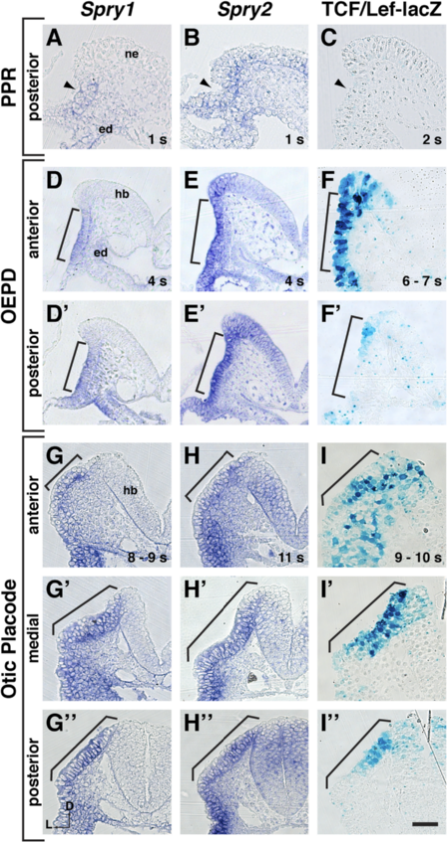
Comparison of *Spry1*, *Spry2*, and Wnt reporter expression domains from PPR to otic placode stages. In situ hybridization analysis of *Spry1* and *Spry2* expression domains compared to Wnt reporter activity in TCF/Lef-lacZ embryos at the stages indicated. Transverse sections are shown, dorsal oriented to the top. **a** – **c** *Spry1* expression, *Spry2* expression, and TCF/Lef-lacZ reporter activity at early somite stages in the posterior PPR. Arrowhead indicates the presumptive otic/epibranchial region. Little or no TCF/Lef-lacZ reporter activity is detected in the posterior PPR at this stage (**c**). (d – f’) *Spry1* expression, *Spry2* expression, and TCF/Lef-lacZ reporter activity in anterior (**d** – **f**) and posterior (d’ – f’) transverse sections through the OEPD. The entire OEPD is bracketed. (g – i”) *Spry1* expression, *Spry2* expression, and TCF/Lef-lacZ reporter activity in anterior (**g** – **i**), medial (g’ – i’), and posterior (g” – i”) transverse sections through the otic placode (bracketed). Abbreviations: neural ectoderm (ne), endoderm (ed), hindbrain (hb). Scale bar, 50 μm

We found that *Spry1* and *Spry2* transcripts were present broadly in the PPR, beginning at pre-somite stages. In the posterior PPR, both genes were expressed strongly in the ectoderm, with additional expression in the underlying mesenchyme and endoderm (Fig. 1a, b). *Spry2* transcript was also detected in neural ectoderm (Fig. 1b). Consistent with previous reports [14, 19], TCF/Lef-lacZ reporter activity was not detected at this stage in the PPR (Fig. 1c). By 3 – 5 s, TCF/Lef-lacZ reporter activity is first detected in the OEPD [14, 19]. At this stage, *Spry1* and *Spry2* transcripts were detected broadly throughout the OEPD in ectoderm, underlying mesenchyme, and endoderm (Fig 1d, d’, e, e’). Whereas expression of *Spry1* and *Spry2* coincided with TCF/Lef-lacZ reporter activity in the anterior ectoderm of the OEPD (Fig. 1d – f), *Spry1* and *Spry2* expression extended further into posterior and ventrolateral regions compared to TCF/Lef-lacZ reporter activity (Fig. 1d’ – f’). In this posterior region, TCF/Lef-lacZ activity was restricted to a few cells adjacent to the developing hindbrain (Fig. 1f’). TCF/Lef-lacZ activity was also detected in migrating neural crest cells, which did not strongly express *Spry1* or *Spry2* (data not shown). When the otic placode became morphologically distinct, *Spry1* and *Spry2* transcripts were detected throughout the otic placode (Fig. 1g – g”, h – h”, brackets). Whereas expression of *Spry1* and *Spry2* was not detected in surface ectoderm anterior or posterior to the placode, expression extended ventrally from the otic placode in epidermal and epibranchial progenitor cells (Fig. 1g – g”, h – h”). Consistent with previous findings [14], Wnt reporter activity was detected in anterior and medial regions of the otic placode, but was absent from a posterior-ventrolateral region (Fig. 1i – i”). In summary, *Spry1* and *Spry2* were expressed in the PPR, prior to the onset of Wnt reporter activity. At the time of onset of Wnt reporter activity in the OEPD and continuing through otic placode stages, *Spry1* and *Spry2* expression domains overlapped with Wnt reporter expression in anterior-medial regions but extended beyond this domain posteriorly and ventrolaterally.

### Expansion of hindbrain *Wnt8a* expression correlated with otic placode expansion

We previously demonstrated that the *Wnt8a* expression domain [14] is expanded in the hindbrain adjacent to the OEPD in *Spry1* and *Spry2* compound mutant embryos (*Spry1*^*−/−*^*; Spry2*^*−/−*^ or “*Spry*-deficient” embryos) at 6 – 7 s, indicating that *Spry* genes regulate Wnt expression [18]. Conversely, hindbrain *Wnt8a* expression is absent in embryos in which *Fgf3* and *Fgf10* have been combinatorially inactivated (*Fgf3*^*−/−*^*; Fgf10*^*−/−*^ mutant embryos and a subset of *Fgf3*^*−/−*^*; Fgf10*^*−/+*^ mutant embryos), as well as in a subset of *Fgf3*^*−/−*^ single mutant embryos [29]. Since the otic placode forms normally in *Fgf3*^*−/−*^ single mutants [29, 38–41], the absence of *Wnt8a* expression in a subset of these mutant embryos is consistent with the finding that *Wnt8a* alone is not required for formation of the otic placode [30].

**Fig. 2:**
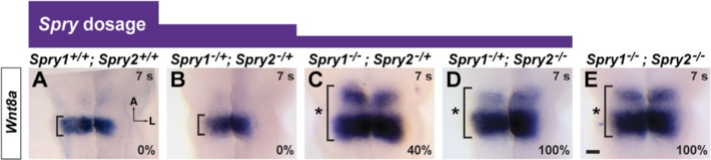
*Wnt8a* expression in a *Spry* gene dosage series. In situ hybridization analysis of *Wnt8a* expression in embryos in which *Spry1* and *Spry2* genes have been combinatorially inactivated. **a** – **e** *Wnt8a* expression in the hindbrain is indicated (brackets) in dorsal views with anterior to the top. Expansions of gene expression domains are highlighted (asterisk). For each genotype, the percentage of embryos with expanded *Wnt8a* expression domains is indicated. Scale bar, 100 μm

We have shown that generation of a *Spry* gene dosage series by combinatorial inactivation of *Spry1* and *Spry2* results in enlargement of the otic placode in a subset of *Spry1*^*−/−*^*; Spry2*^*−/+*^ and *Spry1*^*−/+*^*; Spry2*^*−/−*^ mutant embryos [42]. The penetrance of otic placode enlargement increased with increasing loss of *Spry* gene function, such that 33 – 50 % of *Spry1*^*−/−*^*; Spry2*^*−/+*^ mutant embryos, 71 – 89 % of *Spry1*^*−/+*^*; Spry2*^*−/−*^ mutant embryos, and 83 – 100 % of *Spry*-deficient (*Spry1*^*−/−*^*; Spry2*^*−/−*^) embryos had otic placode phenotypes [42]. To determine whether, as in *Fgf3*^*−/−*^ mutant embryos, otic placode phenotypes are de-coupled from the size of the *Wnt8a* expression domain, we examined *Wnt8a* expression in the *Spry* gene dosage series. We performed in situ hybridization on whole mount embryos to detect *Wnt8a* transcript at 6 – 8 s, prior to the stage when expansion of the otic placode was observed [18, 42]. Consistent with our previous findings, in 100 % of *Spry*-deficient embryos, *Wnt8a* expression in the hindbrain was expanded compared to wild-type controls (compare Fig. 2a to e, 6 out of 6 *Spry1*^*−/−*^*; Spry2*^*−/−*^ double mutant embryos, *n* = 10 *Spry1*^*+/+*^*; Spry2*^*+/+*^ controls). In 100 % of *Spry1*^*−/+*^*; Spry2*^*−/−*^ embryos (Fig. 2d, 5 out of 5 embryos) and 40 % of *Spry1*^*−/−*^*; Spry2*^*−/+*^ embryos (Fig. 2c, 2 out of 5 embryos) *Wnt8a* expression in the hindbrain was expanded. No expansion of *Wnt8a* expression was observed in *Spry1*^*−/+*^*; Spry2*^*−/+*^ compared to wild-type embryos (compare Fig. 2a and b, 0 out of 4 embryos). Thus, in a *Spry* gene dosage series, the frequency at which expanded *Wnt8a* expression was observed was consistent with the frequency at which otic placode expansion was observed. To confirm that enlargement of the otic placode correlated with enlargement of *Wnt8a* expression, we performed in situ hybridization on 6 – 8 s whole mount embryos using a mixture of a *Wnt8a* and *Pax8* probes to visualize the *Wnt8a* expression domain and the otic placode in the same embryo. For each genotype in the *Spry* gene dosage series, if the otic placode appeared larger, the *Wnt8a* expression in the hindbrain also appeared expanded. Conversely, if the otic placode appeared a normal size, the *Wnt8a* expression domain also appeared normal (data not shown, *n* = 3 embryos for each of the following genotypes: *Spry1*^*−/+*^*; Spry2*^*−/+*^ control embryos, *Spry1*^*−/−*^*; Spry2*^*−/+*^ embryos, *Spry1*^*−/+*^*; Spry2*^*−/−*^ embryos, and *Spry1*^*−/−*^*; Spry2*^*−/−*^ double mutant embryos).

Therefore, in *Spry* mutant embryos, unlike *Fgf* mutants, the size of the otic placode correlated with the size of the *Wnt8a* expression domain. Furthermore, *Fgf* genes have differential roles in *Wnt8a* regulation, with *Fgf3* playing a more significant role than *Fgf10* [29]. In contrast, *Spry1* and *Spry2* regulation of both *Wnt8a* expression and otic placode size may be more functionally redundant.

### Inactivation of *Spry1* and *Spry2* did not affect expression of other genes encoding Wnt ligands or Fzd receptors

**Fig. 3:**
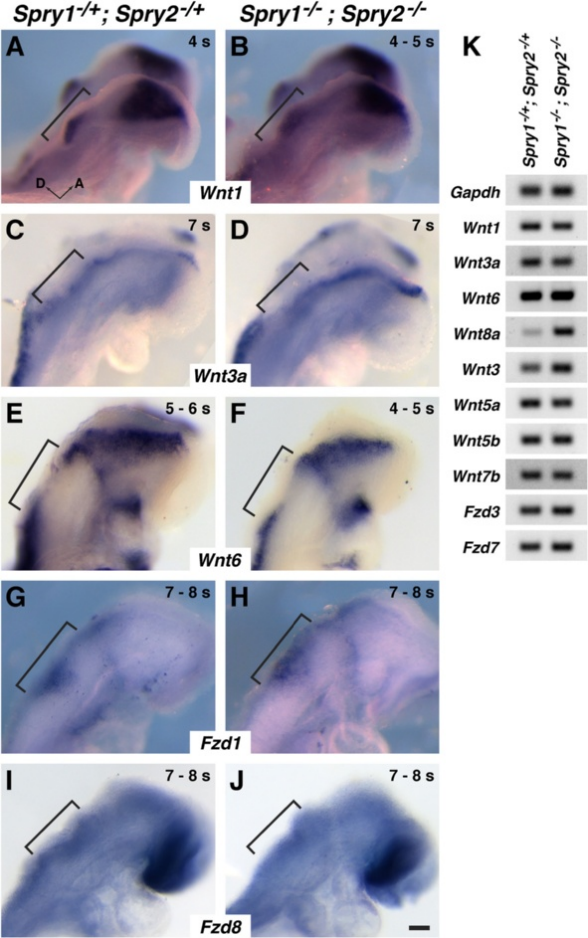
Expression patterns of genes encoding Wnt ligands and receptors are unchanged in *Spry1* ^*−/−*^ *; Spry2* ^*−/−*^ embryos. **a** – **j** In situ hybridization analyses of genes encoding Wnt ligands and receptors in *Spry1* ^*−/−*^ *; Spry2* ^*−/−*^ embryos and controls at OEPD or early otic placode stages. Lateral views of whole-mount embryos are shown; embryos are oriented as indicated. Pre-otic regions are bracketed. **k** Semi-quantitative reverse-transcriptase PCR analyses of candidate *Wnt* and *Fzd* transcript levels in RNA collected from 5 – 7 s hindbrain and OEPD-containing tissue microdissected from the genotypes indicated. *Wnt* genes (*Wnt1*, *Wnt3a*, *Wnt6*, and *Wnt8a*) with known expression adjacent to the OEPD are controls. Scale bar, 100 μm

To determine whether *Spry* genes regulate the expression of other *Wnt* genes besides *Wnt8a* or genes that encode Wnt receptors, we determined the expression of known hindbrain-expressed Wnt genes and OEPD-expressed Frizzled (*Fzd*) genes in *Spry*-deficient and control embryos. Prior to otic placode formation, *Wnt1* (Fig. 3a), *Wnt3a* (Fig. 3c), and *Wnt6* (Fig. 3e) are expressed adjacent to the OEPD in dorsal neural ectoderm, in the region of pre-migratory neural crest cells [19, 29]. We found no gross difference in expression patterns of these genes in *Spry*-deficient embryos compared to control embryos, and minor differences in expression domain were not consistent between genotypes (compare Fig. 3a, c, e to Fig. 3b, d, f; *Wnt1*, *n* = 6 *Spry1*^*−/−*^*; Spry2*^*−/−*^ mutant embryos; *Wnt3a*, *n* = 9 *Spry1*^*−/−*^*; Spry2*^*−/−*^ mutant embryos; *Wnt6*, *n* = 4 *Spry1*^*−/−*^*; Spry2*^*−/−*^ mutant embryos). In addition, genes encoding two Wnt receptors, *Fzd1* and *Fzd8*, are expressed in pre-otic tissue at 6 – 8 s ([29], Fig. 3g, h). No gross difference in the expression of *Fzd1* or *Fzd8* was found in *Spry*-deficient embryos compared to controls (compare Fig. 3g, i to Fig. 3h, j; *Fzd1*, *n* = 6 *Spry1*^*−/−*^*; Spry2*^*−/−*^ mutant embryos, *Fzd8*, *n* = 7 *Spry1*^*−/−*^*; Spry2*^*−/−*^ mutant embryos).

Furthermore, we microdissected tissue containing the OEPD and adjacent neural ectoderm from 5 – 7 s *Spry*-deficient and control embryos. We assessed expression levels of additional candidate *Wnt* and *Fzd* genes expressed in neural ectoderm using semi-quantitative reverse transcriptase PCR (RT-PCR). As confirmation of the microdissection technique and consistent with in situ hybridization analyses, RT-PCR indicated that *Wnt1*, *Wnt3a* and *Wnt6* expression levels were unaffected in *Spry*-deficient embryos (Fig. 3k). Furthermore, consistent with in situ hybridization results, RT-PCR suggested that expression of *Wnt8a* was elevated in *Spry*-deficient embryos (Fig. 3k). We examined additional candidate *Wnt* and *Fzd* genes that have neural ectoderm or otic expression at E8.5 or E9.5: *Wnt3* [1], *Wnt5a* [1, 3, 5], *Wnt5b* [5], *Wnt7b* [1], *Fzd3* [11], and *Fzd7* [11]. Expression levels of all of these genes were comparable in *Spry*-deficient and control embryos (Fig. 3k). Thus, of the genes that encode Wnt ligands and receptors known to be expressed in or adjacent to the OEPD, only *Wnt8a* expression is *Spry*-regulated.

### Wnt signaling activity was increased in *Spry1*^*−/−*^*; Spry2*^*−/−*^ mutants

**Fig. 4:**
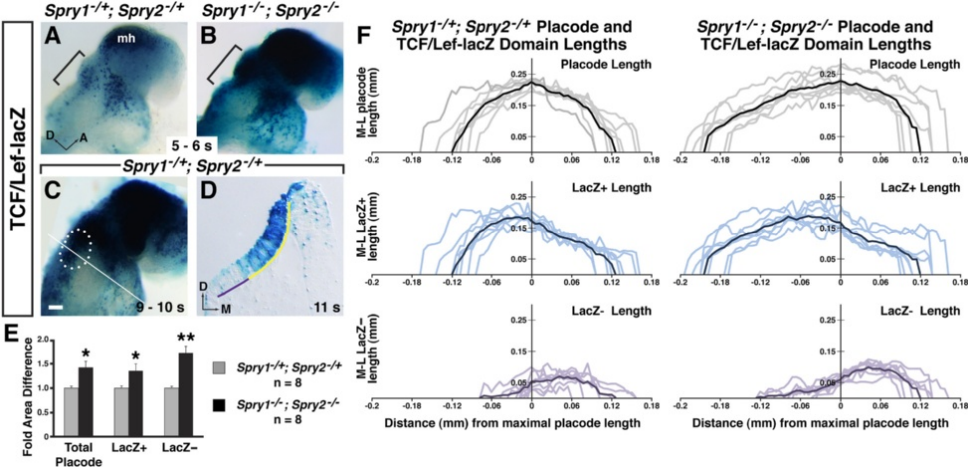
Wnt reporter activity in *Spry1* ^*−/−*^ *; Spry2* ^*−/−*^ mutant and control embryos at OEPD and otic placode stages. **a**, **b** Wnt reporter activity in *Spry1* ^*−/+*^ *; Spry2* ^*−/+*^ control and *Spry1* ^*−/−*^ *; Spry2* ^*−/−*^ mutant embryos. The OEPD region is bracketed; mid-hindbrain region, (mh). Lateral views of whole-mount embryos are shown. **c** Wnt reporter activity in a *Spry1* ^*−/+*^ *; Spry2* ^*−/+*^ control embryo. The otic placode is outlined (white dots). The plane of section shown in (**d**) is indicated with a white line. **d** Transverse section through the otic placode in a *Spry1* ^*−/+*^ *; Spry2* ^*−/+*^ control embryo. An example of the location from which LacZ+ placode lengths were measured is shown with a yellow line. An example of the location from which LacZ- placode lengths were measured is shown with a purple line. Total placode lengths represent the sum of LacZ+ and LacZ- length measurements. **e** Average fold basal area difference between *Spry1* ^*−/+*^ *; Spry2* ^*−/+*^ control and *Spry1* ^*−/−*^ *; Spry2* ^*−/−*^ mutant embryos at 10 – 13 s. Basal area for each placode was calculated as the sum of length measurements multiplied by the thickness of each section. Individual area measurements were normalized by the average area measurement in *Spry1* ^*−/+*^ *; Spry2* ^*−/+*^ controls. *, *p* < 0.05; ** *p* < 0.001. **f** Graphical representation of medial-to-lateral total placode, LacZ+, and LacZ- lengths of the same embryos represented in (**e**). Measurements for each individual otic placode are shown: total placode lengths are shown in grey, LacZ+ lengths in light blue, and LacZ- lengths in light purple. For each genotype, measurements from individual otic placodes were aligned by the maximal total placode length, represented by “0” on the x-axis. The average total placode length, LacZ- length, and LacZ- length are shown with a darker line. Scale bar (**a** – **c**), 100 μm

To determine whether cellular response to Wnt signaling was affected in *Spry*-deficient embryos, we bred the TCF/Lef-lacZ transgene into the *Spry* mutant background, and assayed for lacZ reporter activity in *Spry1*^*−/−*^*; Spry2*^*−/−*^*; TCF/Lef-lacZ/+* mutant embryos compared to *Spry1*^*−/+*^*; Spry2*^*−/+*^*TCF/Lef-lacZ/+* controls. In control embryos, at the onset of TCF/Lef-lacZ reporter activity in the OEPD at 3 – 6 s, reporter-positive cells were detected in an anterior-dorsal domain (Fig. 4a, *n* = 7). In *Spry1*^*−/−*^*; Spry2*^*−/−*^*; TCF/Lef-lacZ/+* mutant embryos TCF/Lef-lacZ reporter activity initiated in the OEPD at the same time as controls, but was qualitatively elevated (Fig. 4b, *n* = 7). In addition, more cells in the OEPD were reporter-positive, such that individual reporter-positive cells could not be easily distinguished from neighboring reporter-positive cells. The increase in TCF/Lef-lacZ reporter activity in the *Spry*-deficient background was not specific to the OEPD: all TCF/Lef-lacZ-positive domains in the whole mount embryo, including the mid-hindbrain (Fig. 4a, b) and posterior embryonic regions (not shown) stained darker and larger in *Spry1*^*−/−*^*; Spry2*^*−/−*^*; TCF/Lef-lacZ/+* mutant embryos. However, the gross pattern of the TCF/Lef-lacZ-positive domain was similar in *Spry*-deficient embryos compared to controls. At the otic placode stage (9 – 13 s), TCF/Lef-lacZ staining was also darker and broader in *Spry1*^*−/−*^*; Spry2*^*−/−*^*; TCF/Lef-lacZ/+* mutant embryos compared to controls (see Additional file 1: Figure S1C, D; *n* = 3 mutant embryos, *n* = 2 controls). These data indicate that Wnt signaling activity is elevated in *Spry*-deficient embryos in both the OEPD and otic placode.

### Effect of loss of *Spry* function on size of the Wnt-reporter-positive and Wnt-reporter-negative domains

In the otic placode, TCF/Lef-lacZ reporter activity is detected in an anterior-dorsal domain but is absent from a posterior-ventrolateral region ([14], Fig. 4c). In *Spry*-deficient embryos, the otic placode is increased in size ([18]). To determine whether the size of the TCF/Lef-lacZ reporter domain increased in proportion with the entire otic placode in *Spry*-deficient embryos, we performed a morphometric analysis. We cut transverse sections of 9 – 13 s *Spry1*^*−/−*^*; Spry2*^*−/−*^*; TCF/Lef-lacZ/+* mutant embryos and *Spry1*^*−/+*^*; Spry2*^*−/+*^*TCF/Lef-lacZ/+* controls that had been stained for LacZ activity. After photography of each section through the otic placode, the medial-lateral lengths of the total placode, LacZ-positive (Fig. 4d, yellow line), and LacZ-negative (Fig. 4d, purple line) regions were measured by tracing along the basal surface of the otic placode using ImageJ. Basal surface area for each region (LacZ-positive, LacZ-negative, or total placode) was calculated by summing all basal length measurements obtained from each section and multiplying this sum by the thickness of the sections (6 μm).

In *Spry*-deficient embryos, the mean basal surface area of the total otic placode was 1.42-fold larger than the area in control embryos (Fig. 4e, 0.055 ± 0.013 mm^2^ in *Spry*-deficient embryos compared to 0.039 ± 0.005 mm^2^ in control embryos, *p* = 0.006, *n* = 8 placodes for each genotype). For the LacZ-positive, Wnt-responsive domain, the mean basal surface area in *Spry*-deficient embryos was 1.36-fold larger than the LacZ-positive area in controls (Fig. 4e, 0.043 ± 0.012 mm^2^ in *Spry*-deficient embryos compared to 0.032 ± 0.004 mm^2^ in control embryos, *p* = 0.022). In contrast, the mean basal surface area of the LacZ-negative domain in *Spry*-deficient embryos was 1.72-fold larger than the LacZ-negative domain in control embryos (Fig. 4e, 0.012 ± 0.003 mm^2^ in *Spry*-deficient embryos compared to 0.007 ± 0.001 mm^2^ in control embryos, *p* = 0.0001). Thus, in *Spry*-deficient embryos, the region of the otic placode with no Wnt reporter activity increased in size to a greater extent than the Wnt-responsive region. As a result, the LacZ-negative domain occupied 18 % of the otic placode in control embryos and 22 % of the otic placode in *Spry*-deficient embryos.

To graphically represent the size and shape of the otic placode, we plotted medial-lateral length measurements from anterior to posterior. We aligned measurements from different individual placodes of the same genotype by the maximal medial-lateral length, a measurement that can be unequivocally identified (Fig. 4f, top graphs, maximal medial-lateral length denoted as “0” and designated as the y-intercept). Graphical representation revealed a shape change in *Spry*-deficient otic placodes. We found that the increased size of the otic placode in *Spry*-deficient embryos (see Fig. 4e) was primarily due to an anterior-posterior expansion, rather than medial-lateral (Fig. 4f, top graphs). The average anterior-posterior length of the otic placode in *Spry*-deficient embryos was 0.319 ± 0.034 mm compared to 0.240 ± 0.027 mm in control embryos (*p* = 0.0002, *n* = 8 placodes for each genotype). Furthermore, in control embryos, the anterior and posterior regions of the otic placode were distributed symmetrically on either side of the widest point (Fig. 4e, top left graph). In contrast, in *Spry*-deficient embryos, the otic placode was asymmetrically shaped around the widest point, with anterior regions elongated.

Measurements of the LacZ-positive and LacZ-negative domains were graphed to align with total placode plots. Thus, for each placode, plots of total placode, LacZ-positive and LacZ-negative measurements were all aligned from the same point of maximal medial-lateral total placode width. In *Spry*-deficient embryos, the shape of the Wnt-responsive, LacZ-positive domain followed the shape of the otic placode, and was more elongated anteriorly (Fig. 4f, middle graphs). Consistent with a disproportionate enlargement of the LacZ-negative domain in *Spry*-deficient embryos, the LacZ-negative domain appeared larger and extended further anteriorly than the LacZ-negative domain in control embryos (Fig. 4f, bottom graphs). The anterior-posterior length of the LacZ-negative domain was 0.200 ± 0.033 mm in *Spry*-deficient embryos compared to 0.135 ± 0.028 mm in controls, *p* = 0.0009). Together, these data suggest that in *Spry*-deficient embryos the otic placode is larger, due to a combination of two factors: 1) an elongation of Wnt-reporter-positive, anterior regions of the placode and 2) an enlargement of a posterior-ventrolateral, Wnt-reporter-negative domain, which showed a greater magnitude increase in size compared to Wnt-reporter-positive regions.

### *Spry* genes function to limit Wnt signaling activity during early inner ear development

Since both *Wnt8a* expression and TCF/Lef-lacZ reporter activity were elevated in *Spry*-deficient embryos, we hypothesized that *Spry1* and *Spry2* function by limiting Wnt signaling activity in approximately 80 % of the otic placode where TCF/Lef-lacZ reporter is active. To functionally test this hypothesis, we genetically reduced Wnt signaling levels in *Spry*-deficient embryos by crossing-in a null allele of the gene that encodes for β-catenin, a Wnt signaling effector (*Spry1*^*−/−*^*; Spry2*^*−/−*^*; β-catenin*^*−/+*^ embryos, referred to below as “experimental” embryos). Littermate *Spry*-deficient embryos, with wild-type gene dosage of *β-catenin* (*Spry1*^*−/−*^*; Spry2*^*−/−*^*; β-catenin*^*+/+*^ embryos, referred to as “*Spry*-deficient controls”) were used as reference controls of *Spry*-deficient otic phenotypes. Littermate *Spry*-heterozygous (*Spry1*^*−/+*^*; Spry2*^*−/+*^*; β-catenin*^*+/+*^) and *Spry-β-catenin*-triple heterozygous (*Spry1*^*−/+*^*; Spry2*^*−/+*^*; β-catenin*^*−/+*^) embryos (referred to below as “heterozygous controls”) were indistinguishable from wild-type embryos and from each other (data not shown) and were used as normal reference controls.

**Fig. 5:**
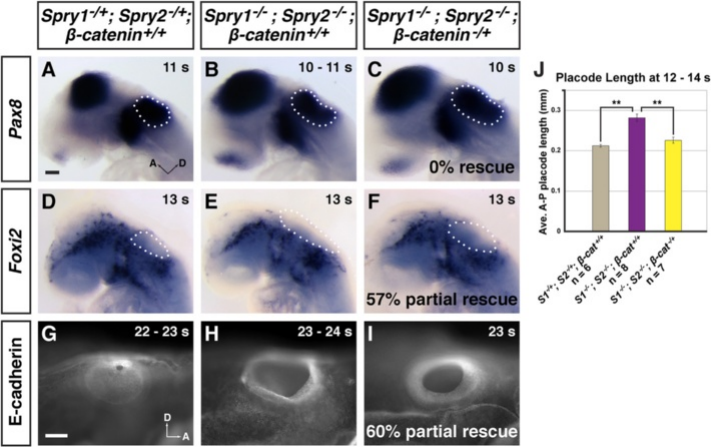
Partial rescue of otic phenotypes *Spry1* ^*−/−*^ *; Spry2* ^*−/−*^ mutants by reducing the dosage of *β-catenin*. **a** – **c** In situ hybridization analysis to detect *Pax8* expression in the otic placode (outlined with white dots). **c** No rescue of otic placode expansions were observed in *Spry1* ^*−/−*^ *; Spry2* ^*−/−*^ *; β-catenin* ^*−/+*^ embryos, as indicated. **d** – **f** In situ hybridization analysis to detect *Foxi2* expression in epidermal/epibranchial cells surrounding the otic placode. The *Foxi2*-negative, otic region is outlined with white dots. **f** A *Spry1* ^*−/−*^ *; Spry2* ^*−/−*^ *; β-catenin* ^*−/+*^ embryo in which the *Foxi2* expression pattern appeared more similar to normal control embryos (**d**), rather than *Spry*-deficient embryos (**e**). The percentage of *Spry1* ^*−/−*^ *; Spry2* ^*−/−*^ *; β-catenin* ^*−/+*^ embryos with partial rescue of the *Foxi2* expression pattern is indicated. **g** – **i** E-cadherin antibody stain on whole-mount embryos to reveal the extent of closure of the otic cup. **i** A *Spry1* ^*−/−*^ *; Spry2* ^*−/−*^ *; β-catenin* ^*−/+*^ embryo in which the otic cup is more closed than any *Spry*-deficient control (see H). The percentage of *Spry1* ^*−/−*^ *; Spry2* ^*−/−*^ *; β-catenin* ^*−/+*^ embryos in which otic cup closure was partially rescued is indicated. **j** Average anterior-posterior lengths. Only the subset of *Spry1* ^*−/−*^ *; Spry2* ^*−/−*^ *; β-catenin* ^*−/+*^ embryos in which *Foxi2* expression domains appeared more similar to normal were selected for length measurement. Scale bar (**a** – **f**), (**g** – **i**), 100 μm

The earliest otic phenotype that we observed in *Spry*-deficient embryos was an enlargement of the otic placode [18]. To visualize the otic placode in experimental, *Spry*-deficient, and heterozygous control embryos, we examined expression of *Pax8*, an early marker of the otic placode [20], in 9 – 11 s embryos. In experimental embryos, the domain of *Pax8* expression in the otic placode looked just as enlarged as in *Spry*-deficient controls (compare Fig. 5c with Fig. 5a and b; *n* = 4 experimental embryos, *n* = 5 *Spry*-deficient control embryos, *n* = 7 heterozygous controls). Other genes whose expression patterns mark the otic placode were not suitable for our analysis: the expansion of the *Pax2* expression domain in the otic placode in *Spry*-deficient embryos is incompletely penetrant [18]; expression of *Hmx3* is reduced in *Spry*-deficient embryos [42]; and *Dlx5* expression is tightly regulated by Wnt signaling [43]. Thus, we examined the expression pattern of *Foxi2*, which is expressed in cranial epidermis, but is excluded from the otic placode [44]. At 12 – 14 s, all *Spry*-deficient controls (*n* = 6) had enlarged *Foxi2*-negative domains, suggesting the presence of an enlarged otic placode (compare Fig. 5e to Fig. 5d; *n* = 18 heterozygous controls). In 57 % (4/7) of experimental embryos, the *Foxi2*-negative domains were more similar in size to heterozygous controls (compare Fig. 5f to Fig. 5d). Whereas in 43 % (3/7) of experimental embryos, the *Foxi2*-negative domains appeared more similar in size to *Spry*-deficient controls (data not shown). To directly examine whether the size of the otic placode was rescued in experimental embryos, we embedded and sectioned embryos to visualize the otic placode anatomically as a thickened epithelium that does not express *Foxi2*. We measured the anterior-posterior length of the otic placode – a metric that was increased in *Spry*-deficient embryos (see Fig. 4f) – by multiplying the number of sections containing an otic placode by the thickness of each section (10 μm). Only the subset of experimental embryos in which the *Foxi2*-negative domain appeared similar to heterozygous controls by whole-mount in situ hybridization was analyzed. Average anterior-posterior lengths of the otic placode in experimental embryos were comparable to lengths in heterozygous controls (Fig. 5j, *n* = 7 experimental placodes, *n* = 6 heterozygous placode, *p* = 0.12), indicating restoration of otic placode expansions in this subset of experimental embryos. In both experimental and heterozygous embryos, average anterior-posterior otic placode lengths were significantly smaller than lengths in *Spry*-deficient controls (Fig. 5j, *n* = 8 *Spry*-deficient placodes, *p* < 0.001 for both comparisons). Thus, by assessment of the *Foxi2*-negative domain and direct measurement of the otic epithelium in histological sections, the otic placode/cup size is partially rescued in experimental embryos by 12 – 14 s. The lack of rescue of the otic *Pax8* expression domain in 9 – 11 s experimental embryos was not investigated further, but suggests the possibility that formation of the early otic placode is less sensitive to *β-catenin* gene dosage.

To examine morphogenesis of the otic epithelium, we stained whole mount embryos with E-cadherin antibody. Consistent with our previous observations [18], in heterozygous controls, by 19 s the rim of the otic cup appeared constricted and in the process of closure and by 24 s the otic cup was completely closed (Fig. 5g, *n* = 10). In contrast, in *Spry*-deficient controls, at 19 s, the otic cup was wide open, with no indication of closure. By 21 – 22 s, otic cup closure initiated, but remained open past 26 s (Fig. 5h, *n* = 10). In 60 % (3/5) of experimental embryos, E-cadherin staining revealed that closure of the otic cup had progressed further than any *Spry*-deficient control embryo examined, but was not as fully closed as in heterozygous controls (Fig. 5i). In the remaining 40 % (2/5) of experimental embryos, the otic cup appeared more similar to *Spry*-deficient embryos.

Finally, we investigated whether incomplete rescue of *Spry*-deficient phenotypes was due to variability in reduction of Wnt signaling in embryos missing one functional allele of *β-catenin*. We produced embryos that were either wild-type or heterozygous for *β-catenin*, which also carried the TCF/Lef-lacZ reporter allele (*β-catenin*^*+/+*^*; TCF/Lef-lacZ/+* and *β-catenin*^*−/+*^*; TCF/Lef-lacZ/+* embryos) and measured Wnt reporter activity. We found that Wnt signaling was reduced in *β-catenin*^*−/+*^*; TCF/Lef-lacZ/+* embryos, but to a variable extent. Whereas in *β-catenin*^*+/+*^*; TCF/Lef-lacZ/+* control embryos, reporter activity was high in the majority of embryos (see Additional file 2: Figure S2A, D), in *β-catenin*^*−/+*^*; TCF/Lef-lacZ/+* embryos, reporter activity was variable, ranging from low to high (Additional file 2: Figure S2B - D). This variability in Wnt signaling activity in embryos missing one functional allele of *β-catenin* could contribute to the incompletely penetrant rescue of *Spry-*deficient phenotypes.

Combined, these data suggest that reduction of *β-catenin* gene dosage in *Spry1*^*−/−*^*; Spry2*^*−/−*^ mutant embryos can partially rescue the otic placode/cup defects observed. These data provide strong genetic evidence that *Spry* regulation of Wnt signaling levels during early inner ear development is functionally significant.

## Discussion

### Timing and nodes of cross talk between FGF and Wnt signaling during otic fate specification

Timing and pattern of *Spry* gene expression and Wnt reporter activity are consistent with the interpretation that FGF signaling precedes and is active in more pre-otic cells. Substantial evidence indicates that FGF signaling is required at multiple steps during the determination of otic fate, beginning with the specification of the PPR, from which all cranial placodes are derived [2, 4, 6, 8]. In multiple organisms, expression of Sprouty genes are induced by FGF signaling, and serves as a read-out of FGF signaling activity [45–48]. This appears to be the case during specification of otic placode fate: *Spry1* gene expression is absent in the dorsal OEPD in *Fgf3*^*−/−*^*; Fgf10*^*−/−*^ double mutants [29], and *Spry1* and *Spry2* expression in the chick is induced by the addition of FGF to early ectodermal cultures [49]. Our data that *Spry1* and *Spry2* are expressed continuously throughout PPR, OEPD and otic placode stages is consistent with the interpretation that FGF signaling functions during each of these stages. Furthermore, FGF signaling levels must be precisely modulated by SPRYs or other negative regulators of RTK signaling, such as the dual-specificity phosphates [50, 51], for proper otic specification [15, 18, 25].

Based upon the activity of the TCF/Lef-lacZ Wnt signaling reporter, it is thought that Wnt signaling is required after specification of the OEPD in the posterior PPR, to influence otic vs. epidermal cell fate decisions [14]. We demonstrate that Wnt reporter activity was detected in a subset of the *Spry*-expression domain, beginning at the OEPD stage. When cellular response to FGF signaling was upregulated in *Spry*-deficient embryos, both the size of the Wnt reporter expression domain and intensity of Wnt reporter staining were increased in the OEPD (see Fig. 4a – b). We found that *Wnt8a* expression was expanded in the hindbrain adjacent to the OEPD in *Spry*-deficient embryos [18], as well as in embryos in which *Spry1* and *Spry2* have been combinatorially inactivated (see Fig. 2). However none of the other genes that encode Wnt ligands or receptors that are known to be expressed at the OEPD stage – *Wnt1*, *Wnt3*, *Wnt3a*, *Wnt5a*, *Wnt5b*, *Wnt6*, *Wnt7b*, *Fzd1*, *Fzd3*, *Fzd7*, and *Fzd8* – were up-regulated in *Spry*-deficient embryos. The expansion and increase in Wnt reporter activity in *Spry*-deficient embryos could be due to the expansion of *Wnt8a* expression alone. Alternatively, the increase in Wnt reporter activity could also be due to changes at other points in the Wnt signaling pathway.

Cross talk between the Wnt and FGF signaling pathways can occur downstream of receptor activation at common target proteins, or nodes (see [52] for review). One such node is GSK3β, which phosphorylates cytoplasmic β-catenin to target it for proteasome-mediated degradation [53]. Wnt signaling inactivates GSK3β via phosphorylation, allowing β-catenin to enter the nucleus, bind members of the TCF/Lef family of transcription factors, and activate transcription of target genes (see [54–57] for recent reviews). FGF signaling can also inactivate GSK3β via phosphorylation by AKT [53, 58]. Thus, by activation of the PI3K-AKT cascade, leading to inactivation of GSK3β, FGF signaling can induce nuclear translocation of β-catenin. Inhibition of *Sprouty* gene function can lead to increased levels of phosphorylated AKT in certain cellular contexts [59], suggesting that in the inner ear, it may be possible for synergy between the Wnt and FGF signaling pathways to occur through phosphorylation of GSK3β. In addition to its transcriptional function, β-catenin is a component of the adherens junction, where it links cadherins to the actin cytoskeleton [60]. In the mouse primitive streak, *Fgfr1* indirectly promotes Wnt signaling by downregulating E-cadherin expression through expression of the Snail transcriptional repressor [61]. Reduced levels of E-cadherin allow for increased amounts of β-catenin to translocate to the nucleus and activate Wnt target genes. However, by immunohistochemistry, we did not detect a dramatic reduction of E-cadherin staining in the otic placode/cup in *Spry*-deficient embryos (see Fig. 5h and data not shown). Thus, it is unlikely that increased FGF signaling in *Spry*-deficient embryos relieves E-cadherin-mediated sequestration of β-catenin in the cytoplasm, thus promoting Wnt signaling. Finally, cross talk between the FGF and Wnt signaling pathways can occur at the level of transcription. In *Drosophila*, the ETS-domain transcriptional repressor Anterior open (Aop, or Yan) competes with the ETS-domain transcriptional activator Pointed (Pnt) to repress gene expression downstream of RTKs [62]. Phosphorylation by MAPK leads to activation of Pnt and inactivation of Aop, resulting in target gene expression [63]. In addition, Aop represses gene expression downstream of Wingless (Wg, the Wnt-1 ortholog), through interaction with Armadillo (Arm, the β-catenin ortholog) [64], leading to transcriptional repression of Wg pathway components [65]. In the *Drosophila* tracheal system, inactivation of Aop by MAPK leads to de-repression of both FGF and Wnt-regulated genes, thus integrating both FGF and Wnt signaling at the level of a single transcriptional repressor [66]. Thus, it is possible that a variety of cellular targets downstream of FGF signaling can also cross talk with the Wnt signaling pathway to ultimately increase TCF/Lef-lacZ reporter activity in *Spry*-deficient embryos.

### FGF and Wnt signaling cooperate during early inner ear development

When FGF signaling levels are elevated in a *Spry* gene dosage series, expansion of the otic placode and the *Wnt8a* expression domain correlate. The functional significance of this correlation is unclear, since in the mouse, *Wnt8a* is not required for otic placode formation, although it may play a role in combination with multiple *Wnt* genes [30]. However, in multiple organisms ectopic expression of Wnt can induce the formation of ectopic or enlarged otic vesicles [16, 21, 23, 27]. In some cases, ectopic induction of otic tissue by Wnt overexpression may be indirect: early over-expression of Wnt leads to posteriorization of the embryo, leading to ectopic expression of both Fgf genes and otic competence factors [21, 23]. Later activation of Wnt signaling, during specification of the otic placode, leads to expansion of the otic domain at the expense of neighboring epidermal or epibranchial cells [14, 16]. Thus, it is possible that in *Spry*-deficient embryos, elevated *Wnt8a* expression, in combination with elevated response of tissue to FGF signaling due to loss of SPRY negative regulation, is sufficient to ectopically direct more cells to an otic fate. Furthermore, the correlation between expansion of the otic placode and expansion of the *Wnt8a* hindbrain expression domain in a *Spry* gene dosage series is consistent with the possibility that FGF and Wnt signaling cooperate during otic placode formation.

We demonstrate that cross talk between the FGF/SPRY and Wnt signaling pathways is functionally required by performing a dominant genetic interaction experiment. Genetic reduction of *β-catenin* gene dosage in *Spry*-deficient embryos (*Spry1*^*−/−*^*; Spry2*^*−/−*^*; β-catenin*^*−/+*^ embryos) resulted in partial restoration of the normal *Foxi2* expression pattern and otic placode size. This indicates that otic (*Foxi2*-negative) vs. epidermal (*Foxi2*-positive) cell fate decisions occur more normally in *Spry*-deficient embryos in which *β-catenin* levels are reduced than in *Spry*-deficient embryos alone. Furthermore, closure of the otic cup was partially rescued in *Spry*-deficient embryos in which *β-catenin* levels were genetically reduced. The delay in otic cup closure observed in *Spry*-deficient embryos could be due to the increased size of the otic placode and/or defects in morphogenesis of the otic cup during invagination [18, 42]. Similarly, increased size of the otic placode and defects in otic cup closure are observed in embryos in which an activated mutant form of *β-catenin* is expressed using the *Pax2-Cre* driver [14]. Thus, partial genetic rescue of the otic cup closure defect in *Spry*-deficient embryos missing one copy of the *β-catenin* gene suggest that *Spry* genes and *β-catenin* function in the same cellular processes that lead to proper closure of the otic cup. Combined, the genetic rescue observed suggests that *β-catenin* and *Spry* genes function in the same genetic pathway during early inner ear development, and demonstrate that aspects of FGF/SPRY signaling in the otic placode are mediated, directly or indirectly, by β-catenin.

### FGF-independent characteristics of the Wnt signaling response

Although *Spry* genes and *β-catenin* function in the same genetic pathway in early inner ear development, multiple characteristics of Wnt reporter activity are independent of *Spry* gene function. First, in *Spry*-deficient embryos, the timing of onset of Wnt reporter activity was unaffected. Thus, elevated response of tissue to FGF does not generate a precocious response to Wnt signals. The timing of onset of Wnt signaling may be controlled independently of FGF signaling. This possibility is consistent with the finding that expression of the genes that encode candidate Wnt ligands and receptors are unaffected in *Spry*-deficient embryos (*Wnt1*, *Wnt3*, *Wnt3a*, *Wnt5a*, *Wnt5b*, *Wnt6*, *Wnt7b*, *Fzd1*, *Fzd3*, *Fzd7*, and *Fzd8*) or *Fgf*-deficient embryos (*Wnt1*, *Wnt3a*, *Wnt6* [29]). Alternatively, onset of Wnt signaling may be controlled upstream of FGFR activation, by the spatial distribution and activity of FGF ligands. The finding that *Wnt8a* expression is reduced or absent in embryos in which *Fgf3* and *Fgf10* have been inactivated is consistent with this possibility [29].

Another characteristic of the Wnt signaling response that is independent of *Spry* gene function is the spatial distribution of the Wnt responsive domain. In the OEPD and otic placode, Wnt reporter activity was detected in anterior and dorsal regions, but was absent from a posterior-ventrolateral region. Although the Wnt reporter domain was larger in *Spry*-deficient embryos, it was still localized to anterior and dorsal regions. Thus, the spatial localization of Wnt ligands and receptors must be unaffected by increased tissue-response to FGF. Interestingly, although the size of the Wnt reporter domain increased in *Spry*-deficient embryos, the size of the reporter-negative domain increased even more (1.72-fold for the reporter-negative domain vs. 1.36-fold for the reporter-positive domain). Therefore, in *Spry*-deficient embryos, the entire otic placode is larger due to increased response of pre-otic ectoderm to FGF. However, expansion of the Wnt-reporter-positive domain is more constrained than the Wnt-reporter-negative region. One possible explanation for the size constraint of the Wnt-reporter-positive domain is that multiple signals, including FGF and Wnt, are required for otic fate specification in this domain and the effective amount of these signals is limiting. Stringent cross talk between the FGF and Wnt signaling pathways may be one mechanism that ensures that otic fate specification in the anterior and dorsal domains is tightly regulated. For the posterior-ventrolateral domain, the effective amount of signals, including FGF, that regulate otic specification must be less stringently controlled. Alternatively, intrinsic anterior-posterior differences may affect the ability of the pre-otic ectoderm to respond to FGF and Wnt inductive signals. These differences may be influenced by other signaling pathways such as retinoic acid [67] or BMP [68].

The role of FGF signaling in the otic placode may be analogous to its role in the developing vertebrate limb bud. During limb development, FGFs, expressed in the apical ectodermal ridge (AER), have dual functions: 1) to specify the initial progenitor pool for all of the skeletal elements of the limb and 2) in conjunction with other signaling pathways, to expand these progenitor pools, with distal elements more sensitive to loss of AER-FGF function than proximal skeletal elements [69, 70]. Similarly, in the otic placode, the FGF/SPRY pathway is required for specification of all otic progenitors. However, response of different regions of the otic placode to increased FGF signaling differs, and may depend on differential requirements for other signaling pathways, including Wnt. Recently, genomic approaches have been completed to identify FGF-regulated genes in the otic placode [29, 49]. To fully understand heterogeneity of the otic progenitor domain, the challenge will be to comprehensively define the distinct phenotypic responses and transcriptional targets that are activated and repressed by FGF alone, Wnt alone, and combinations of these and other signaling molecules during specification and patterning of the otic placode.

## Conclusions

In this study, we investigated cross talk between FGF and Wnt signaling during specification and early morphogenesis of the otic placode. We show that FGF signaling precedes and is active in more cells of the OEPD and otic placode than Wnt signaling. Furthermore, we provide in vivo evidence that FGF signaling activates the Wnt signaling pathway upstream of TCF/Lef transcriptional activation. FGF regulation of the Wnt signaling pathway is functionally relevant, since early inner ear defects in *Spry*-deficient embryos can be genetically rescued by reducing the gene dosage of *β-catenin*. Thus, as in chick, *Xenopus*, and zebrafish, FGF and Wnt signals cooperate during otic specification in the mouse. However, we found that in *Spry*-deficient embryos, the Wnt-reporter-positive domain increases in size, but not to the same extent that the Wnt-reporter-negative domain expands. This result suggests that although otic specification is globally regulated by FGF signaling, otic specification of cells in which both FGF and Wnt signaling are active is more tightly regulated.

## Additional files

Additional file 1: Figure S1. Wnt reporter activity in *Spry1* ^*−/−*^ *; Spry2* ^*−/−*^ mutant and control embryos at otic placode stages. Representative mutant and control embryos that have been incubated with X-gal either overnight (A, B) or for 90 mins. (C, D) are shown. Brackets indicate the otic placode region. (PNG 1346 kb) Additional file 2: Figure S2. Wnt reporter activity in embryos missing one functional copy of *β-catenin*. Lateral views of *β-catenin* ^*+/+*^ *; TCF/Lef-lacZ/+* control embryos (A) and *β-catenin* ^*−/+*^ *; TCF/Lef-lacZ/+* embryos (B, C) stained for TCF/Lef-lacZ reporter activity for 60 mins. For each embryo, staining intensity was scored as low, medium, or high. (D) Staining intensity tallies for each genotype. (PNG 996 kb)

## Abbreviations

FGF: Fibroblast growth factor
GSK3β: glycogen synthase kinase 3β
MO: Morpholino
OEPD: Otic-epibranchial progenitor domain
PPR: Pan-placodal region
RTK: Receptor tyrosine kinase
Spry: Sprouty
