# Supplementary figures and images for: Cooperative and independent functions of FGF and Wnt signaling during early inner ear development
Source: BMC Dev Biol. 2015 Oct 6;15:33. doi: 10.1186/s12861-015-0083-8 (PMC4594887; doi:10.1186/s12861-015-0083-8)

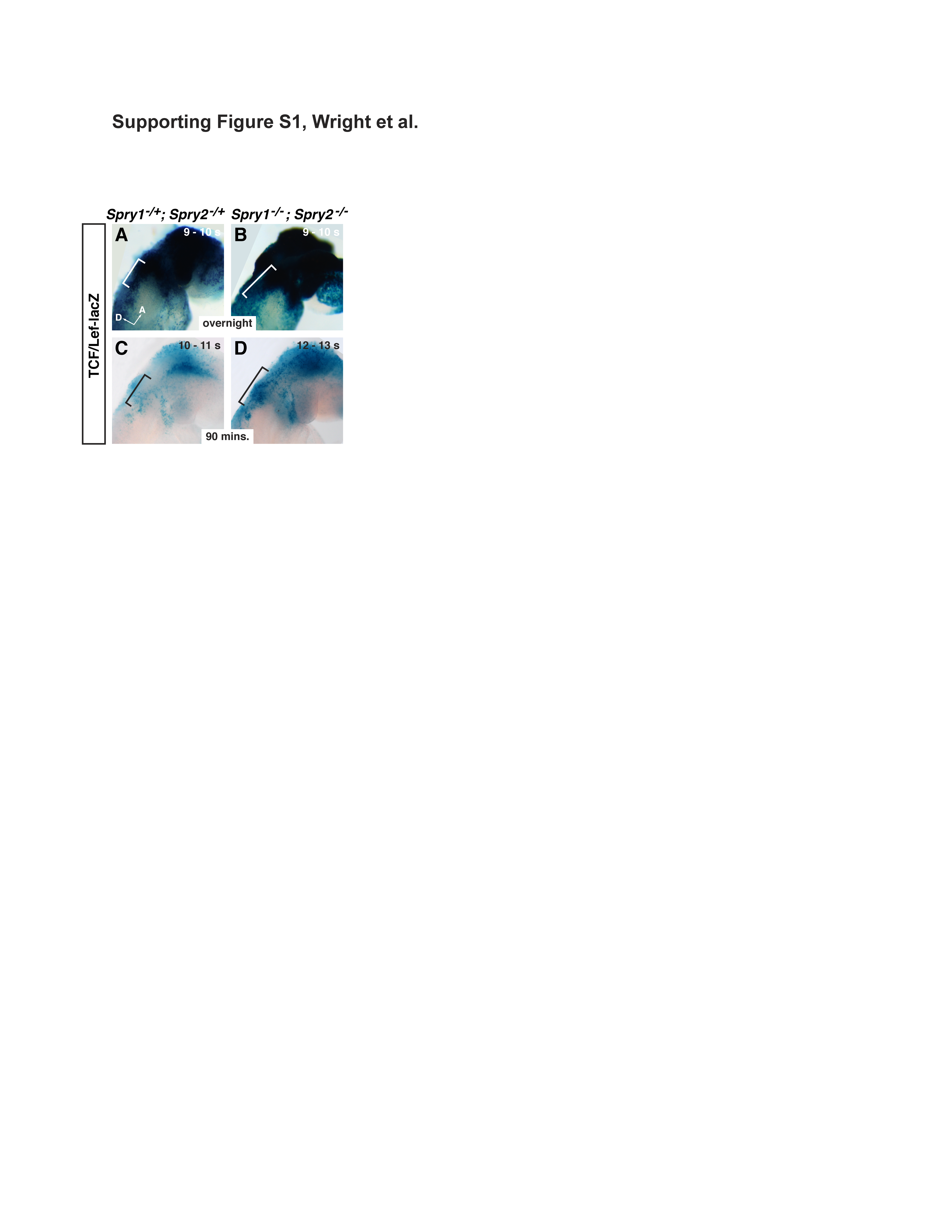

Supplement: Additional file 1: Figure S1. — Wnt reporter activity in Spry1 −/− ; Spry2 −/− mutant and control embryos at otic placode stages. Representative mutant and control embryos that have been incubated with X-gal either overnight (A, B) or for 90 mins. (C, D) are shown. Brackets indicate the otic placode region. (PNG 1346 kb) [file 12861_2015_83_MOESM1_ESM.png]
